# Prevalence of priapism in individuals with sickle cell disease and implications on male sexual function

**DOI:** 10.31744/einstein_journal/2020AO5070

**Published:** 2020-04-16

**Authors:** Mateus Andrade Alvaia, Heros Aureliano Antunes da Silva Maia, Alana de Medeiros Nelli, Carina Oliveira Silva Guimarães, Evanilda Souza de Santana Carvalho, José Murillo Bastos Netto, Eduardo de Paula Miranda, Cristiano Mendes Gomes, José de Bessa

**Affiliations:** 1 Universidade Estadual de Feira de Santana Feira de SantanaBA Brazil Universidade Estadual de Feira de Santana, Feira de Santana, BA, Brazil.; 2 Universidade Federal de Juiz de Fora Juiz de ForaMG Brazil Universidade Federal de Juiz de Fora, Juiz de Fora, MG, Brazil.; 3 Universidade Federal do Ceará FortalezaCE Brazil Universidade Federal do Ceará, Fortaleza, CE, Brazil.; 4 Hospital das Clínicas Faculdade de Medicina Universidade de São Paulo São PauloSP Brazil Hospital das Clínicas, Faculdade de Medicina, Universidade de São Paulo, São Paulo, SP, Brazil.

**Keywords:** Anemmia, sickle cell, Priapism/epidemiology, Erectile dysfunction, Sexuality

## Abstract

**Objective:**

To evaluate epidemiological aspects of priapism in patients with sickle cell disease, and these aspects impact on adult sexual function.

**Methods:**

This was a cross-sectional study including individuals with sickle cell disease who were evaluated at a reference center for sickle cell. Participants completed a structured questionnaire about their sociodemographic characteristics and priapism events. Sexual function was assessed using validated two instruments, the Erection Hardness Score and one about the sex life satisfaction.

**Results:**

Sixty-four individuals with median aged of 12 (7 to 28) years were interviewed. The prevalence of priapism was 35.9% (23/64). The earliest priapism episode occurred at 2 years of age and the latest at 42 years. The statistical projection was that 71.1% of individuals of the study would have at least one episode of priapism throughout life. Patients with episodes of priapism (10/23) had significantly worse erectile function Erection Hardness Score of 2 [1-3]; p=0.01 and were less satisfied with sexual life 3 [3-5]; p=0.02.

**Conclusion:**

Priapism is usually present in childhood, and severe episodes are associated with cavernous damage, impairment in the quality of the erection, and lower sexual satisfaction.

## INTRODUCTION

“Sickle cell disease” (SCD) is a generic term that encompasses a group of hereditary hemolytic anemia characterized by structural changes in the beta hemoglobin’s chain, leading to the production of abnormal hemoglobin, which is called HbS.^1 , 2^ This disease is recognized by the World Health Organization (WHO) as a serious global public health problem, with great impact on the morbidity and mortality of the affected population.^3^ It affects mainly Afro-descendants, and its pathophysiology is related to the occurrence of vaso-occlusive episodes, in small vessels, leading to the signs and symptoms of the disease.^2^ The estimated prevalence of SCD in Brazil is 25,000 to 50,000 individuals, with the incidence of 1 case to every 650 live births in Northeastern regions and 1 to every 1,300 in the South region of the country.^4^

Priapism is a clinical disorder characterized by prolonged penile erection in the absence of sexual interest or desire.^5^ This is a urologic emergency as it damages the erectile tissue and may lead to loss of functional erection.^6^ Priapism affects all age groups of patients with SCD, and this is more common in older patients. The incidence is as high as 3.6% among teenagers (<18 years of age) and increases to up to 42% in adult patients.^7^

Priapism is classified according to the degree of blood oxygenation in the corpora cavernosa as low flow (ischemic) and high flow (non-ischemic) priapism. Initially described in 1934, ischemic priapism is the most typical form of priapism in patients with SCD, and it may present as an acute event or as recurrent episodes.^8 , 9^ The precise pathophysiological mechanism of priapism in patients with SCD is still unclear;^10^ however, it has been suggested that blood drainage from the penis is compromised as a result of vaso-occlusion of small vessels. Additionally, prolonged episodes lead to persistent ischemia of the erectile tissue, which can lead ultimately to cavernosal fibrosis and persistent erectile dysfunction (ED).^11^

The duration of the episodes represents the most significant predictor for adequate future erectile function. Therefore, interventions should be initiated within 4 to 6 hours, focusing on corpora bodies detumescence, pain reduction, and ED prevention.^12^ Current initial general treatment strategies consist of oxygenation, hydration, and blood transfusions. Patients may require penile aspiration associated with injection of vasoactive agents. For refractory cases, surgical intervention is indicated.^7 , 13 , 14^

To avoid episodes of priapism is the most important strategy to prevent corpora damage and ED. Different strategies are available with conflicting results. Sickle cell disease control using hydroxyurea has been suggested to improve the quality of life of patients with SCD because and reduce the number of vaso-occlusive crises, including priapism.^15^

Priapism in individuals with SCD is prevalent and has negative consequences to one’s quality of life. Few studies have performed an in-depth analysis of the prevalence and consequences of this condition.

## OBJECTIVE

To evaluate the epidemiological aspects of priapism and its impact on the sexual function of adults with sickle cell disease.

## METHODS

### Patient population and definition of priapism

This was an cross-sectional and retrospective study in which all male patients of all ages with SCD followed at our reference center at *Universidade Estadual de Feira de Santana* (Bahia, Brazil) were evaluated between October 2016 and October 2017.

The study was approved by the institutional Ethics Committee under the protocol 1.440.239 (CAAE: 49493315.3.1001.0053), and all study subjects or legal guardians signed informed consent.

Patients were asked to answer a structured questionnaire about their socio-demographic characteristics, their type of sickle hemoglobin disease, and episodes of priapism. It also included questions on their knowledge about priapism and a diary of events, frequency, duration, recurrence, triggers, and treatments.

Priapism was defined as an erection lasting more than 4 hours. Prolonged erections (PE) were defined as an erection lasting >1.5 hour, but less than 4 hours.^3^

### Sexual function assessment

**Figure 1:**
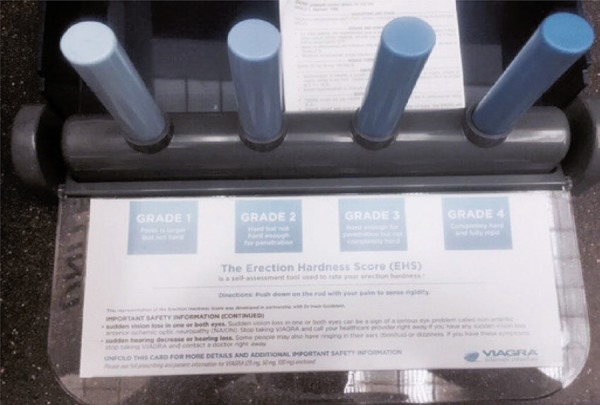
Tool for Erection Hardness Score assessment

Erectile function was evaluated in individuals older than 18 years using the Erection Hardness Score (EHS) scale. Erection Hardness Score was performed using a rigidity assessment device developed by Pfizer ( Figure 1 ).

Patients were instructed to apply pressure using the palm of their hand against four bendable shafts and indicate their best erection rigidity during sexual stimulation, which was classified as follows: grade 1, if increased tumescence but not hard; grade 2, if hard penis but not enough for penetration; grade 3, if enough for penetration but not completely rigid; grade 4, if completely hard and rigid penis.

Satisfaction with sex life (SSL) was also evaluated using a Visual Analog Scale. Patients were asked to choose the option that best suited their reality: very dissatisfied; dissatisfied; almost equally satisfied and dissatisfied; pleased, and very satisfied.

### Statistical analysis

Quantitative, continuous or ordinal variables were described by their central tendency measures (means or medians) and by their respective dispersion measures (standard deviation or interquartile range), while the nominal or qualitative variables by their absolute values, percentages or proportions. The Student’s *t* or the Mann-Whitney tests were used to compare the continuous variables. In the comparison of categorical data, χ^2^ test and its variants were used.

Values of p<0.05 were considered significant. GraphPad Prism, version 8.0.3, GraphPad Software, San Diego, California, USA, was used in the analyzes.

## RESULTS

Sixty-four male subjects aged between 2 and 69 years, with a median age of 12 (7-24) years were evaluated. The most frequent SCD type was hemoglobinopathy type SS (HbSS) in 39 individuals (60.9%), followed by hemoglobinopathy type SC (HbSC) in 19 (29.8%), as indicated in table 1 .

**Table 1:** Types de hemoglobinopathies

Hemoglobinopathy	n (%)
Hemoglobinopathy type SS	39 (60.9)
hemoglobinopathy type SC	19 (29.7)
S/α thalassemia	1 (1.5)
Unknown	5 (7.8)

Forty-six (71.9%) patients were unaware of the meaning of “priapism”. After clarification and explanation about priapism, it was possible to verify a prevalence of 35.9% (23/64) and, of these, 69.6% (16/23) had the HbSS genotype.

**Figure 2:**
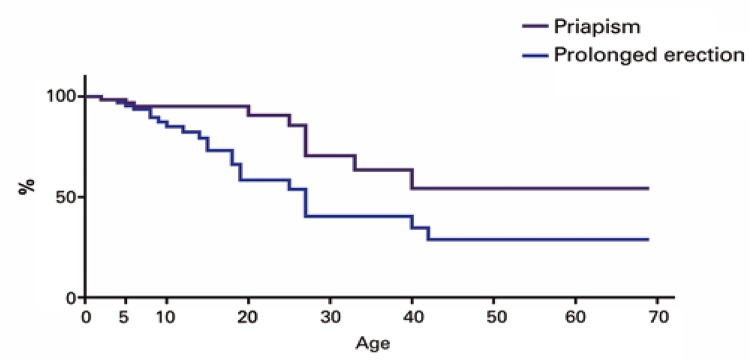
Estimation of complication-free interval regarding priapism and prolonged erection in patients with sickle cell disease

The earliest episode of priapism occurred as early as 2 years of age and the later episode at 42 years. More than two thirds of the patients (16/23; 69.6%) had their first episode before 20 years of age ( Table 2 ). The statistical projection was that 71.1% of the individuals in this sample would have at least one event of PE, and 45.7% of them would present with at least one episode of priapism throughout their lives ( Figure 2 ).

**Table 2:** Time of the priapism episodes and precipitating factors

Data related to underlying disease	n (%)
Time when episodes started	
Morning	7 (30.4)
Afternoon	3 (13.0)
Night	5 (21.8)
During sleep	8 (34.8)
Precipitating factor	
Cold	6 (26.1)
Fever	2 (8.7)
Dehydration	4 (17.4)
Sexual stimuli	4 (17.4)
Others	2 (8.7)
Unknown	5 (21.7)
Age when occurred the first episode of priapism	
0-10	7 (30.4)
10-20	9 (39.1)
20-30	4 (17.4)
>30	3 (13.0)

Of the 23 patients who reported some PE without sexual stimuli, 43.5% (10/23) presented acute priapism events while the remainder had only PE. Of those with acute priapism, 80% (8/10) had HbSS genotype, including a three year-old child. Recurrent episodes of PE were the initial presentation in 78.2% (18/23). Homozygotes presented significantly more episodes of priapism than heterozygotes (odds ratio − OR=2.93; 95% of confidence interval − 95%CI: 1.02-9.04).

Priapism episodes occurred mainly either at night or morning, and cold temperatures were the major precipitating factor. There was no report of previous PDE5i or intracavernous injections use ( Table 2 ). Only 47.8% (11/23) of men who had priapism reported going to the emergency room for medical assistance. Regarding the use of hydroxyurea, only 23.6% (17/64) of the patients had a prescription, accounting for 58.8% (10/17) of those who reported priapism.

**Figure 3:**
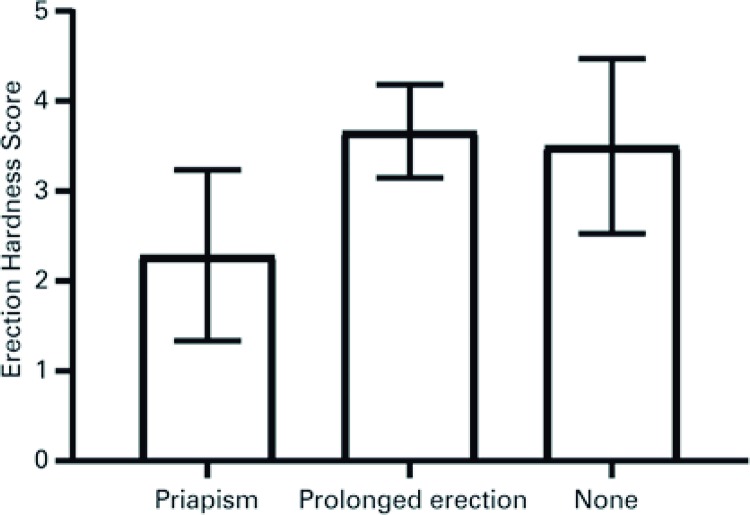
Erection Hardness Scores in patients with sickle cell disease, priapism and prolonged erection

**Figure 4:**
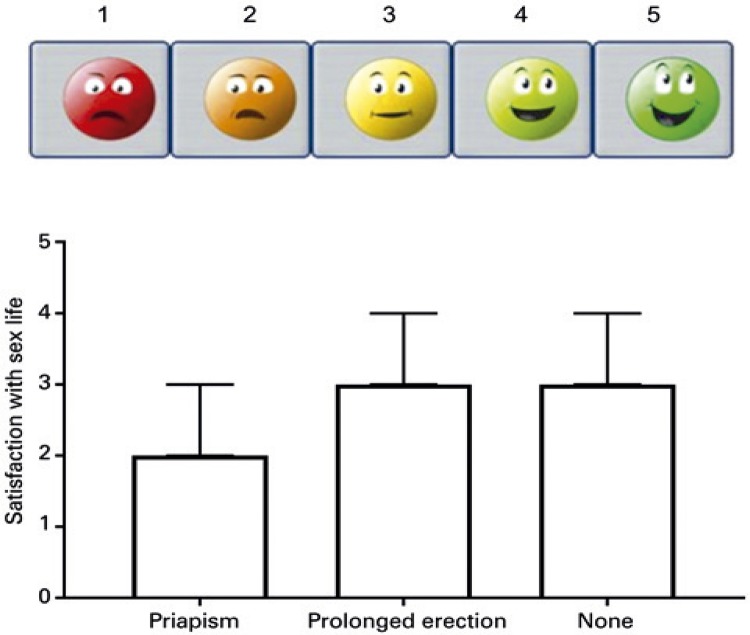
Satisfaction with sex life in the patients with sickle cell disease and priapism or prolonged erection

In the group of the patients older than 18 years of age who were analyzed with EHS and SSL, those with a history of priapism had significantly worse erections: EHS scored 2 [1-3] in comparison to individuals with PE (EHS=4 [3-4]; p=0.01) ( Figure 3 ). Also, the patients who had priapism were less satisfied with their sexual life than the individuals with PE (SSL=2 [1-2]; *versus* 3 [3-5]; p=0.02) ( Figure 4 ).

## DISCUSSION

Blood dyskinesia such as SCD are the main risk factors for developing priapism.^6^ These episodes are usually underestimated, not only because patients do not seek care out of embarrassment, but also as many of them do not see it is as a result of SCD.^16^ In this study, the prevalence of priapism in men with SCD was similar to those found in the literature.^10 , 16 , 17^ The genetic type HbSS was related to episodes of priapism, which is also in accordance with other series.^10 , 18^

Priapism initially manifests itself as minor episodes or PE in childhood and adolescence. However, it may progressively become episodes of priapism.^18^ In an international multicenter observational study, the mean age of onset of priapism episodes was 11 years, with 25% of cases of ischemic priapism presenting during prepubertal years and the chance of having a first-time episode after the third decade of life was extremely low.^9^ In the present study, the median age of men who had priapism was 12 years, and seven patients reported priapism episodes occurred before the age of 10. Of these, three cases were 6 years of age or younger (3, 5 and 6 years of age), and this dramatically increased the chances of developing erectile problems in adulthood. Only three cases presented erectile problems after the age of 30.

Episodes of priapism occur spontaneously, with erections usually at night or when having fever and dehydration.^19^ In this study, nocturnal events were also the most frequent, which were mostly precipitated by cold temperatures. In a national study conducted at the Emergency Department in the United States, however, there was no significant difference in the number of emergency visits following priapism in the coldest seasons of the year.^18^

Priapism in patients with SCD is a urologic emergency that requires immediate intervention to avoid erectile tissue damage.^11 , 20 , 21^ In the present study, 11 patients sought emergency care because of painful and persistent erection. All patients were seen at local public health facilities, as there was no reference center for these emergencies in our region. Current guidelines recommend corpora aspiration and injection of sympathomimetics for the initial management of ischemic priapism, associated with hydration, oxygenation, and systemic alkalinization.^21^ However, prevention of priapism is the most effective way to avoid long-term degeneration of erectile function.

Hydroxyurea is currently proposed as a prophylactic treatment of priapism in patients with SCD due to the consequent elevation of lobin, reduction of neutrophils and reticulocytes, and decrease in cellular adhesion to vascular endothelium.^8 , 10 , 11^ Also, one study suggested a possible effect of hydroxyurea on the recovery of erectile function after the resolution of the event.^11^ Although many patients reported the use of hydroxyurea in this study, about 60% of them had episodes of priapism.

Prolonged erections, not complicated by priapism, tend to resolve itself entirely with no damage to erectile function. Severe episodes have less favorable and less predictable outcome.^16^ Episodes of priapism often lead to erectile tissue necrosis and subsequent fibrosis by fibroblast proliferation. Erectile dysfunction is the most common negative consequence in these populations, since irreversible damage to erectile tissue occurs, with documented rates of up to 90% for priapism lasting longer than 24 hours. However, some reports of ED may also be related to episodes of recurrent PE, in which the duration is inferior to 4 hours.^17^

Together with ED, priapism reverberates in mental health, with feelings of despair, anxiety, embarrassment, isolation, and dissatisfaction with sexual life. Sexual satisfaction is a complex issue, and few studies have approached this tissue among patients with SCD.

This study is not free of limitations. Its retrospective nature may have been under the influence of recall bias. Besides, patients were seen in different non-specialized centers during the acute event with non-standardized treatment protocols.

## CONCLUSION

Episodes of priapism are seen early in life and in the majority of cases in homozygotics hemoglobinopathy type SS. A great number of patients are unaware of the meaning of “priapism” and a few of them seek for medical treatment. Patients who present priapism have a worse erectile function and are less satisfied with their sexual life.
