# Supplementary material for: Prevalence of priapism in individuals with sickle cell disease and implications on male sexual function
Source: Einstein (Sao Paulo). 2020 Apr 16;18:eAO5070. doi: 10.31744/einstein_journal/2020AO5070 (PMC7186002; doi:10.31744/einstein_journal/2020AO5070)
Supplement: Translation

# Translation: Prevalência de priapismo em indivíduos com doença falciforme e suas implicações na função sexual masculina

**Authors:** Mateus Andrade Alvaia, Heros Aureliano Antunes da Silva Maia, Alana de Medeiros Nelli, Carina Oliveira Silva Guimarães, Evanilda Souza de Santana Carvalho, José Murillo Bastos Netto, Eduardo de Paula Miranda, Cristiano Mendes Gomes, José de Bessa

**Affiliations:** 1 Brasil Universidade Estadual de Feira de Santana, Feira de Santana, BA, Brasil.; 2 Brasil Universidade Federal de Juiz de Fora, Juiz de Fora, MG, Brasil.; 3 Brasil Universidade Federal do Ceará, Fortaleza, CE, Brasil.; 4 Brasil Hospital das Clínicas, Faculdade de Medicina, Universidade de São Paulo, São Paulo, SP, Brasil.

**Keywords:** Anemia falciforme, Priapismo/epidemiologia, Disfunção erétil, Sexualidade

## Abstract

**Objetivo:**

Avaliar aspectos epidemiológicos do priapismo em pacientes com doença falciforme e o impacto desses aspectos na função sexual de adultos.

**Métodos:**

Trata-se de estudo transversal, que incluiu indivíduos com doença falciforme acompanhados em um centro de referência. Os participantes responderam a um questionário estruturado acerca das características sociodemográficas e eventos de priapismo. A função sexual foi avaliada por meio de dois instrumentos validados, a Escala de Rigidez de Ereção e um sobre satisfação com a vida sexual.

**Resultados:**

Foram entrevistados 64 indivíduos com média de idade de 12 (7-28) anos. A prevalência de priapismo foi de 35,9% (23/64). O episódio mais precoce ocorreu aos 2 anos de idade e o mais tardio, aos 42 anos. A projeção estatística foi de que 71,1% desses sujeitos teriam pelo menos um episódio de priapismo ao longo da vida. Pacientes adultos com episódios de priapismo (10/23) apresentaram função erétil significativamente pior Escala de Rigidez de Ereção de 2 [1-3]; p=0,01 e estavam menos satisfeitos com a vida sexual 3 [3-5]; p=0,02.

**Conclusão:**

O priapismo manifesta-se desde a infância, e episódios graves estão associados a dano cavernoso, prejuízo na qualidade da ereção e menor satisfação sexual.

## INTRODUÇÃO

“Doença falciforme” (DF) é um termo genérico que engloba grupo de anemias hemolíticas hereditárias, caracterizadas por mudanças estruturais na cadeia beta de hemoglobina, levando à produção de hemoglobina anormal, chamada HbS.^1 , 2^ Essa doença é reconhecida pela Organização Mundial da Saúde (OMS) como um problema de saúde pública global, com grande impacto na morbidade e na mortalidade da população afetada.^3^ A DF acomete principalmente afrodescentes, e sua patofisiologia está relacionada a ocorrência de episódios vaso-oclusivos em pequenos vasos, causando alguns sinais e sintomas da doença.^2^ A prevalência estimada da DF no Brasil é 25 mil a 50 mil indivíduos, com incidência de 1 caso para cada 650 nascidos vivos nas regiões nordestinas e 1 em cada 1.300 no sul do Brasil.^4^

Priapismo é um distúrbio clínico caracterizado por ereção peniana prolongada na ausência de interesse ou desejo sexual.^5^ Trata-se de uma emergência urológica, já que pode causar lesão aos tecidos eréteis e causar perda da função erétil.^6^ O priapismo afeta todos os grupos etários com DF e é mais comum entre pacientes idosos. A incidência é tão alta quanto 3,6% entre adolescentes (<18 anos) e aumenta para até 42% em pacientes adultos.^7^

O priapismo é classificado de acordo com o grau de oxigenação sanguínea no corpo carvernoso como priapismo de baixo fluxo (isquêmico) e de alto fluxo (não isquemico). Inicialmente descrito em 1934, o priapismo isquêmico é a forma mais típica em pacientes com DF e pode apresentar-se como evento ou espisódio recorrente.^8 , 9^ O mecanismo patofisiológico preciso do priapismo em pacientes com DF permanece obscuro,^10^ mas sugere-se que a drenagem sanguínea do pênis é comprometida devido à vaso-oclusão de pequenas veias. Além disso, os episódios prolongados levam à isquemia persistente do tecido erétil, que pode causar fibrose dos tecidos cavernosos e disfunção erétil (DE) persistente.^11^

A duração dos episódios representam o preditor mais significativo para função erétil adequada no futuro. As intervenções devem ser iniciadas de 4 a 6 horas, focando em detumescência do membro, diminuição da dor e prevenção de DE.^12^ Atualmente, as estratégias de tratamento inicial geral consistem em oxigenação, hidratação e transfusões sanguíneas. Os pacientes podem necessitar de aspiração peniana associada com injeção de agentes vasoativos. Para casos refratários, é indicada intervenção cirúrgica.^7 , 13 , 14^

Evitar episódios de priapismo é um estratégia importante para prevenir a lesão do corpo e a DE. Diferentes estratégias de tratamentos estão disponíveis, mas com resultados controversos. O controle da doenças falciforme por meio da hidroxiurea tem sido sugerido para melhorar a qualidade de vida dos pacientes com DF devido à redução do número de crises vaso-oclusivas, incluindo o priapismo.^15^

Os priapismo em indivíduos com DF é prevalente e tem consequências negativas para qualidade de vida. Poucos estudos têm sido conduzidos incluindo análises profundas da prevalência e consequências desta condição.

## OBJETIVO

Avaliar aspectos epidemiológicos e seu impacto na função sexual de adultos com doenças falciforme.

## MÉTODOS

### População de pacientes e definição do priapismo

Estudo tranversal e restrospectivo incluindo pacientes do sexo masculino de diversas faixas etárias, que foram acompanhados e avaliados entre outubro 2016 e outubro de 2017 em um centro da Universidade Estadual de Feira de Santana (Bahia, Brasil).

Os pacientes foram questionados sobre características sociodemográficas, tipos de DF, e episódios de priapismo. Além disso, foram incluídas questões sobre conhecimento sobre priapismo, existência de diário de eventos, frequência, duração, recorrência, estímulos, e tratamento.

Priapismo foi definido como um ereção por mais de 4 horas. Ereções prolongadas (EP) foram definidas como uma ereção por >1,5 hora, mas por menos de 4 horas.^3^

### Avaliação da função sexual

A DE foi avaliada em indivíduos com mais de 18 anos utilizando Escala de Rigidez de Ereção (ERE). A ERE foi realizada por meio de dispositivo para avaliação de rigidez desenvolvido pela Pfizer ( Figura 1 ).

**Figura 1:** Ferramenta para avaliação da Escala de Rigidez de Ereção

Pacientes foram instruídos a aplicar pressão com palma da mão contra uma barra dobrável e indicar a rigidez de sua ereção durante o estímulo sexual, que foi classificado como grau 1, se aumento de tumescência, mas não rígido; grau 2, se pênis rígido, mas não o suficiente para penetração; grau 3, se suficiente para penetrar, mas não completamente rígido; e grau 4, se completamente ereto e rígido.

A satisfação com a vida sexual (SVS) também foi avaliada por meio de Escala Visual Analógica. Os pacientes foram solicitados a escolher a opção que melhor se adequasse à sua realidade: muito insatisfeito, insatisfeito, nem satisfeito nem insatisfeito, satisfeito e muito satisfeito.

### Análise estatística

Variáveis quantitativas, contínuas ou ordinais foram descritas por mediadas centrais tendenciosas (médias e medianas) e por respectiva dispersão de medidas (desvio padrão ou variação interquatil), enquanto as variáveis foram descritas como nominais ou qualitativas, por meio de valores absolutos, porcentagens ou proporções. O teste *t* de Student ou o teste de Mann-Whitney foram utilizados para comparar as variáveis contínuas. Na comparação de dados categóricos, o teste χ^2^ e suas variáveis foram utilizadas.

Os valores de p<0,05 foram considerados significantes. Para as análises, adotou-se o GraphPad Prism, versão 8.0.3, GraphPad Software, San Diego, Califórnia, EUA.

O estudo foi aprovado pelo Comitê de Ética institucional, protocolo 1.440.239 (CAAE: 49493315.3.1001.0053), e todos os participantes ou responsáveis legais assinaram o Termo de Consentimento.

## RESULTADOS

Foram avaliados 64 homens, entre 2 e 69 anos, com média de idade de 12 (7-24) anos. O tipo de DF mais frequente foi hemoglobinopatia SS (HbSS), encontrado em 39 pacientes (60,9%), seguido de hemoglobinopatia SC (HbSC), encontrado em 19 pacientes (29,8%), como indicado na tabela 1 .

**Tabela 1:** Tipos de hemoglobinopatias

Hemoglobinopatia	n (%)
Hemoglobinopatia SS	39 (60,9)
Hemoglobinopatia SC	19 (29,7)
S/α talassemia	1 (1,5)
Desconhecida	5 (7,8)

Um total de 46 (71,9%) pacientes desconhecia o significado do termo “priapismo”. Após esclarecimento e explicação sobre termo, foi possível verificar prevalência de 35,9% (23/64) e, destes, 69,6% (16/23) tinham genótipo HbSS.

O mais precoce de episódio de priapismo ocorreu em pacientes com menos de 2 anos de idade e o episódio mais tardio, aos 42 anos. Mais de dois terços dos pacientes (16/23; 69,9%) tiveram seu primeiro episódio antes dos 20 anos de idade ( Tabela 2 ). A projeção estatística foi de que 71,1% dos indivíduos na amostra tinham pelo menos um evento de EP, e 45,7% deles apresentariam pelo menos um episódio de priapismo ao longo da vida ( Figura 2 ).

**Tabela 2:** Tempo de episódios de priapismo e fatores de precipitação

Dados relacionadas à doença subjacente	n (%)
Periódio em que os episódios iniciaram	
Manhã	7 (30,4)
Tarde	3 (13,0)
Noite	5 (21,8)
Durante o sono	8 (34,8)
Fatores de precipitação	
Resfriado	6 (26,1)
Febre	2 (8,7)
Desidratação	4 (17,4)
Estímulo sexual	4 (17,4)
Outros	2 (8,7)
Desconhecido	5 (21,7)
Idade quando ocorreu o primeiro episódio de priapismo	
0-10	7 (30,4)
10-20	9 (39,1)
20-30	4 (17,4)
>30	3 (13,0)

**Figura 2:** Estimativa de intervalo livre de complicação em relação ao priapismo e à ereção prolongada em pacientes com doença falciforme

Dos 23 pacientes que relataram algum tipo de EP sem estímulo sexual, 43,5% (10/23) apresentaram eventos de priapismo agudo, enquanto o restante teve somente EP. Daqueles com priapismo agudo, 80% (8/10) tiveram genótipo HbSS, incluindo uma criança de 3 anos. Os episódiso recorrentes de EP foram identificados em 78,2% (18/23) dos participantes. Homozigotos apresentaram significativamente mais episódios de priapismo do que heterozigotos ( *odds ratio* − OR=2,93; intervalo de confiança de 95% − IC95%: 1,02-9,04).

Os episódios de priapismo ocorreram principalmente no período noturno ou diurno durante temperaturas baixa, sendo esse o principal fator de precipitação. Não houve relato de PDE5i anterior ou uso de injeções intracarvenosas ( Tabela 2 ). Somente 47,8% (11/23) dos homens que apresentaram priapismo relataram procurar aconselhamento médico. Em relação ao uso de hidroxiurea, somente 23,6% (17/64) dos pacientes tiverem a prescrição, contabilizando 58,8% (10/17) daqueles que relataram priapismo.

No grupo de pacientes com mais de 18 anos de idade, que foram avaliados com ERE e SVS, aqueles com história de priapismo tiveram ereções significativamente piores: ERE pontuou 2 [1-3] em comparação a indivíduos com EP (ERE=4 [3-4]; p=0,01) ( Figura 3 ). Pacientes que tiveram priapismo estavam menos satisfeito com sua vida sexual do que indivíduos EP (SVS=2 [1-2] *versus* 3 [3-5]; p=0,02) ( Figura 4 ).

**Figura 3:** Escala de Rigidez de Ereção em pacientes com doença falciforme, priapismo e ereção prolongada

**Figura 4:** Satisfação com vida sexual em pacientes com doença falciforme, priapismo ou ereção prolongada

## DISCUSSÃO

Disquinesia tal como a DF são fatores de risco para desenvolvimento priapismo.^6^ Esses episódios são geralmente subestimados, não apenas porque os pacientes não procuravam ajuda médica devido ao constrangimento, mas também porque muitos indivíduos não observam os episódios como secundário à DF.^16^ Neste estudo, a prevalência de priapismo em pacientes homens com DF foi similar aquela encontrada na literatura.^10 , 16 , 17^ O tipo de genética HbSS foi relacionada a episódios de priapismo, que também está de acordo com outras séries de estudo.^10 , 18^

Iniciamente, o priapismo manifesta-se como episódios menores ou EP na infância e adolescência. Porém, pode progressivamente tornar-se episódios de priapismo.^18^ Em estudo internacional observacional multicêntrico, a média de idade de episódios de priapismo foi de 11 anos, com 25% dos casos de priapismo isquêmico apresentados durante os anos de pré-puberdade, e a chance de ter primeiro episódio após terceira década de vida foi extremamente baixa.^9^ Em nosso estudo, a média de idade dos homens que possuíam priapismo foi 12 anos. Além disso, sete pacientes relataram episódios de priapismo ocorrendo antes dos 10 anos de idade. Dentre os casos, três pacientes tinham 6 anos de idade ou eram mais jovens (3, 5 e 6 anos), e os episódios aumentaram dramaticamente as chances de desenvolvimento de problemas erétil na vida adulta. Em apenas três casos, os episódios de priapismo se iniciaram após 30 anos de idade.

Os episódios de priapismo ocorreram espontaneamente, com ereções em geral noturnas ou quando paciente apresentava febre ou desidratação.^19^ Neste estudo, os eventos noturnos também foram mais frequentes, e a maioria foi precipitada por temperaturas frias. Em estudo conduzido no Departamento de Emergência Nacional nos Estados Unidos, porém, há diferença significante em número de consultas emergenciais seguidas de priapismo em períodos de estações mais frias no ano.^18^

O priapismo em paciente com doenças falciforme é uma emergência urológica que requer intervenção imediata, para evitar lesão no tecido erétil.^11 , 20 , 21^ No presente estudo, 11 pacientes procuraram emergência devido à ereção persistente e dolorosa. Todos receberam assistência em instituição de saúde pública, já que, na região onde este estudo foi conduzido, não há centro de referência para tais emergências. As diretrizes atuais recomendam aspiração do corpo e injeção de simpatomiméticos, para manejo inicial de priapismo isquêmico, associadas a hidratação, oxigenação e alcalinização sistêmica.^21^ Porém, a prevenção de priapismo é o modo mais efetivo de evitar degeneração de longo prazo da função erétil.

Atualmente, a hidroxiurea é a proposta de tratamento profilático para priapismo em pacientes com DF, devido à consequente elevação de hemoglobina fetal, à redução de neutróficos e reticulócitos, e à diminuição da adesão celular ao endotélio vascular.^8 , 10 , 11^ Além disso, um estudo sugeriu hidroxiurea como possível causa de recuperação da função erétil após a resolução de evento.^11^ Apesar de muitos pacientes relatarem o uso de hidroxiurea no estudo, cerca de 60% deles tiveram episódios de priapismo.

Ereções prolongadas, não complicadas pelo priapismo, tendem a se resolverem completamente, sem lesão da função erétil. Os episódios graves têm menos resultados favoráveis e preditivos.^16^ Episódios de priapismo, em geral, levam à necrose do tecido erétil e à fibrose subsequente, por proliferação por fibroblasto. A DE é a consequência negativa mais comum nessas populações, desde ocorrências de lesões irreversíveis ao tecido erétil com taxas documentadas de até 90% para priapismo por mais de 24 horas. Contudo, alguns relatos de DE podem também estar relacionados a episódios de EP recorrente, em que a duração é inferior a 4 horas.^17^

Junto da DE, o priapismo afeta a saúde mental e leva a sentimentos de desespero, ansiedade, constrangimento, isolamento e insatisfação com vida sexual. A satisfação sexual é um questão complexa, e poucos estudos têm abordado a questão entre pacientes com DF.

Nosso estudo não está livre de limitações. A natureza restropectiva do nosso relato pode estar sobre influência de viés de recordação. Além disso, não se observaram pacientes em diferentes centros não especializados durante o evento agudo e com adoção de protocolos de tratamento não padronizados.

## CONCLUSÃO

Episódios de priapismo ocorrem precocemente na vida e, na maioria dos casos, em homozigotos hemoglobinopatia tipo SS. Um grande número de pacientes desconhece o significado do termo “priapismo”, e poucos procuram tratamento médico. Além disso, os pacientes que apresentam priapismo têm piora na função erétil e são menos satisfeitos com a vida sexual.
